# A novel approach to exploring youth non-suicidal self-injury heterogeneity: individual differential psychopathology network analysis

**DOI:** 10.1186/s12991-025-00606-5

**Published:** 2025-10-17

**Authors:** Zhongliang Jiang, Zhongyi Liu, Qinghao Yang, Wenyan Zhang, Xianbin Wang, Kai Yang, JinHyun Jun, Yonghua Cui, Tianyuan Lei

**Affiliations:** 1 Department of Psychiatry, Beijing Children’s Hospital, Capital Medical University, National Center for Children’s Health, Beijing, China; 2 Laboratory for Clinical Medicine, Capital Medical University, Beijing, 100045 China

**Keywords:** Non-suicidal self-injury, Student, Individual differential psychopathology network, IDPN

## Abstract

**Background:**

Non-suicidal self-injury (NSSI) is a common behavioral problem among children and adolescents. Previous studies of NSSI have been mostly group-based and lacked specific characterization of individuals with NSSI.

**Methods:**

Using convenience sampling, we surveyed all students from three junior high schools in a county in China, totaling 2,376 participants (mean age 13.66, *SD* 0.98). Assessments included NSSI, anxiety, depression, personality traits, and family environment. Based on the network template perturbation approach, we employed three steps—constructing the reference network, constructing the perturbed network, and computing the individual differential psychopathology network (IDPN). The IDPN was then constructed from questionnaire scores to capture the degree to which abnormal individuals deviate from the normative level. K-means clustering was then applied to explore the internal heterogeneity of NSSI.

**Results:**

Among 2,376 students, 881 (37.1%) exhibited NSSI. Following IDPN construction, we selected 8 characteristics for clustering analysis based on significant changes in at least 2% of the samples. The elbow method indicated 2 clusters. Fisher discriminant analysis showed a classification accuracy of 95.8%, reflecting a good clustering effect. Severity of NSSI in Group 1 was lower than in Group 2, with scores for 7 out of 8 characteristics also lower in Group 1, except for “Control-Organization.” NSSI was associated with personality traits, depression, and family environment, with stronger connections between individual features linked to higher NSSI severity.

**Conclusion:**

We introduced the concept of IDPN in psychometrics, which can reveal relationships among individual characteristics and identify distinct patient subgroups. Further research is needed to confirm its reproducibility and generalizability.

**Supplementary Information:**

The online version contains supplementary material available at 10.1186/s12991-025-00606-5.

## Introduction

Non-suicidal self-injury (NSSI) refers to the direct and intentional self-infliction of harm to one’s body without suicidal intent [1]. Unlike self-harm, NSSI specifically emphasizes that the individual does not have suicide as the goal of their actions. The commonly accepted purpose of NSSI is to alleviate negative thoughts or feelings or to solve certain problems [2, 3]. Adolescence is a particularly vulnerable period for mental health and behavioral issues, making NSSI highly prevalent during this time [4]. The prevalence of NSSI among children and adolescents is approximately 17%−18% [5, 6], indicating that one in every five to six children or adolescents is affected by NSSI. Individuals who engage in repeated NSSI behaviors may have more psychological development or emotional problems [7], and in severe cases, it can even lead to suicide [8]. Given its high prevalence and the serious harm it causes to individuals, it is crucial to focus on the early identification and intervention of NSSI.

Current research on NSSI broadly categorizes its influencing factors into biological, personal, and environmental factors. Biological factors that may influence NSSI include changes in the function of the hypothalamic-pituitary-adrenal (HPA) axis and the hypothalamic-pituitary-thyroid (HPT) axis [9, 10], as well as abnormalities in the secretion of stress hormones and pain-regulating substances [11, 12]. Among the personal factors influencing NSSI, personality traits have been strongly evidenced [13]. Among various personality trait dimensions, neuroticism has been widely shown to be positively correlated with NSSI [14, 15]. Additionally, some studies had mentioned a negative correlation between extraversion and NSSI [16]. Additionally, the presence of emotional problems such as anxiety and depression can increase the likelihood of NSSI [17, 18]. In environmental factors, children or adolescents with NSSI often come from families with various dysfunctions, such as low cohesion among family members [19], poor emotional expression, and high levels of behavioral control [20]. The manifestations and frequency of NSSI exhibit significant heterogeneity among different individuals. Previous findings on the influencing factors of NSSI provided a reference for the selection of NSSI-related factors in this study. Moreover, previous research on the risk factors of NSSI has mostly focused on population or group levels, with little description of the relationship between NSSI and its risk factors at the individual level. This may result in some risk factors for NSSI being overlooked in studies.

The network analysis method, which is now widely used to describe mental health disorders or abnormal behaviors [21], represents the interconnections between symptoms or nodes at the group level. Its advantages include universality, compatibility, and good visual representation [22]. Normative modeling is a recent statistical method that maps characteristic heterogeneity at the individual level among patients [23, 24]. It provides statistical inferences regarding the extent to which each abnormal individual deviates from the normal pattern and has been successfully applied in the field of mental health [25–27]. A study on a structural brain covariance network [28] based on this technology has provided us with insights on how to construct individual psychological networks by quantifying the deviations in psychological measurement levels between individuals with NSSI and those without. Traditional network analysis methods are typically conducted at the group or population level. When the number of patients within a sample is relatively small, their characteristics are easily obscured by those of the larger control group. This novel network analytic approach addresses this limitation by first constructing a reference network to define the normative group and subsequently generating an individual psychological network for each patient while evaluating the significance of each feature within the network. This approach not only overcomes the limitations of traditional network analysis but also provides new perspectives and observable features for future research, with reduced requirements regarding the distribution of patients and controls within the sample.

Our research aims to establish the individual differential psychopathology network (IDPN) and apply it for the first time to measure NSSI. In this study, we used a sample of 2,376 middle school students. In addition to assessing NSSI, we evaluated 17 influencing factors of NSSI that have been confirmed in previous studies, which were mainly categorized into four domains: anxiety, depression, family environment, and personality traits. We constructed the characteristics of the network by establishing the correlations among these psychological factors. A normative network was constructed using 1,495 samples without NSSI, followed by the establishment of an IDPN for 881 samples with NSSI to identify the characteristics affecting NSSI. After constructing the IDPN, we used the selected abnormal characters for K-means clustering analysis and compared the feature differences between the two groups to explore the heterogeneity of individuals with NSSI.

## Methods and measures

### Participants

Using convenience sampling, three junior high schools in a county in Shandong Province, China, were selected, and a survey was conducted with all students from first to third grade in these schools. To ensure the quality of data collection, teachers responsible for administering the questionnaires received standardized training from psychiatric professionals, which covered topics such as privacy protection and survey procedures. After the survey, all questionnaires were collected, logically reviewed, and sorted, with any invalid questionnaires being excluded. A total of 2,900 questionnaires were distributed, and after information sorting, 2,376 participants’ questionnaires were included in this study. Among the samples, there were 1,136 male participants and 1,240 female participants. Out of the 2,376 samples, a total of 881 samples exhibited NSSI.

This study was approved by the Ethics Committee of Shandong Mental Health Center. Written informed consent was obtained from both the participants and their guardians before the survey.

### Questionnaires

We collected the demographic information of participants using a general information questionnaire. The Adolescents Self-Harm Scale, Self-Rating Anxiety Scale (SAS), Self-Rating Depression Scale (SDS), Neuroticism Extraversion Openness Five-Factor Inventory (NEO-FFI), and Family Environment Scale-Chinese Version (FES-CV) were used to gather information on participants’ NSSI, anxiety, depression, personality traits, and family environment, respectively.

#### General information questionnaire

Using a self-administered general information questionnaire, subjects were surveyed on their general information, including gender, age, and grade level.

#### Adolescents self‑harm scale

The questionnaire was initially developed by Zheng Ying et al. in 2006 to assess NSSI over the past year and is now widely used in NSSI research in China [17, 29]. It mainly investigates 15 common types and frequencies of NSSI. Additionally, there is one open-ended question to supplement any NSSI types and frequencies not included in the listed items. For each NSSI type, the questionnaire assesses the frequency and the subjective judgment of the severity of the harm caused by NSSI. NSSI frequency is assessed on a 4-point scale: 0, 1, 2–4, and ≥ 5 times. Severity is rated on a 5-point scale: none, mild, moderate, severe, and extremely severe, with detailed descriptions of each severity level provided in the questionnaire instructions for the participants to choose from. The total NSSI severity is calculated as the sum of the frequency multiplied by the severity for each of the 16 NSSI types. In this study, the Cronbach’s alpha was 0.88.

#### Self‑rating anxiety scale (SAS)

The SAS [30] is a classic self-rating questionnaire used to assess the anxiety symptoms of participants. It includes 20 items, using a 4-point scoring method (with 5 items being reverse scored). Participants select the option that best describes their situation for each item. The total score of the 20 items is multiplied by 1.25 and then rounded to obtain the standard score of the scale, with higher scores indicating more severe anxiety symptoms. The Cronbach’s alpha was 0.83 [31].

#### Self‑rating depression scale (SDS)

The SDS [32] is a classic self-rating questionnaire used to assess the depressive symptoms of participants. It includes 20 items, using a 4-point scoring method (with 10 items being reverse scored). Participants select the option that best describes their situation for each item. The total score of the 20 items is multiplied by 1.25 and then rounded to obtain the standard score of the scale, with higher scores indicating more severe depressive symptoms. The Cronbach’s alpha was 0.80 [31].

#### Neuroticism extraversion openness five-factor inventory (NEO-FFI)

The NEO-FFI [33] is a self-assessment tool used to measure the personality traits of participants [34]. It consists of 60 items, using a 5-point scoring method (including 27 reverse-scored items), to measure the participant’s five dimensions: neuroticism (N), extraversion (E), openness (O), agreeableness (A), and conscientiousness (C). Higher scores indicate a stronger presence of that trait in the participant. In this study, the Cronbach’s alpha was 0.84.

#### Family environment scale (chinese version, FES-CV)

The FES-CV [35] is a self-assessment questionnaire used to measure the family environment of participants. After revisions by Chinese scholars, it has been widely used in China to assess participants’ family relationships and characteristics [36, 37]. The FES-CV consists of 90 items, each with a binary response option (yes or no), where participants select the option that best reflects their actual situation. The questionnaire measures ten dimensions of the family, with higher scores indicating more prominent features in each dimension. Previous research has demonstrated good reliability and validity of this questionnaire [38, 39]. In this study, the Cronbach’s alpha was 0.61.

### Constructing individual differential psychopathology network (IDPN)

**Fig. 1:**
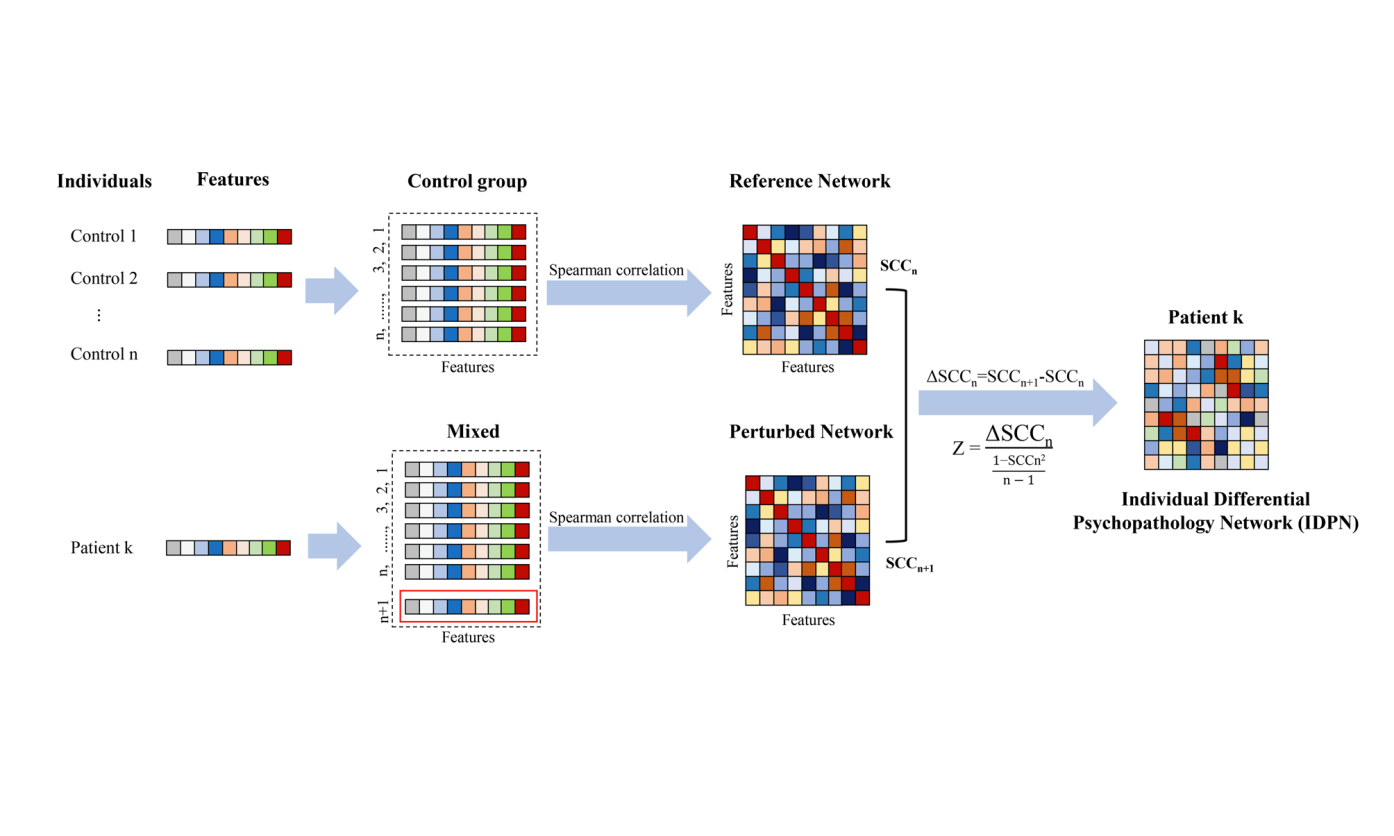
The flowchart of constructing individual differential psychopathology network (IDPN). Note: First, a reference network was constructed using n samples without NSSI. Then, based on this reference network, one sample with NSSI was added to form a perturbed network. By subtracting the reference network from the perturbed network, we obtained the IDPN for this sample with NSSI

Using R (version 4.2.2) to construct IDPN. We referenced the method used to construct the individual differential structural covariance network (IDSCN) for brain functional individual differences [28, 40, 41] and extended its application to the field of psychological measurement, creating the individual differential psychopathology network (IDPN). The method consists of three steps: Step 1, constructing the Reference Network; Step 2, constructing the Perturbed Network; Step 3, computing the IDPN (Fig. 1).

Specifically, we first selected the psychological measurement features to be included in the study. We constructed a Spearman correlation matrix for the psychological measurement features in all control groups (participants without NSSI), forming the Reference Network. In the Reference Network, each cell represents a Spearman correlation coefficient (SCC). We named the Reference Network including all control groups as SCCn. Second, based on the samples from all control groups, we added a patient k to form a new sample and constructed a Spearman correlation matrix, forming the Perturbed Network. We named this network using *n* + 1 samples as SCCn + 1. Subsequently, we calculated the difference between SCCn + 1 and SCCn (ΔSCC). Previous studies have shown that the difference (ΔSCC) between the Perturbed Network and the Reference Network follows a “volcano distribution” [42]. Therefore, the formula for computing the *Z*-score of ΔSCC (based on *Z*-test or *U*-test) is:

$$\text Z =\:\frac{\rm{{\triangle}{SCC}}\text{n}}{\frac{1\text{-SCCn}^{2}}{\text{n}-1}}$$

Therefore, we obtain the IDPN for patient group k. In the IDPN, each cell represents the weight (*Z*-score) of the character between features. Subsequently, we further obtain the *p*-values for each character based on its *Z*-score (using Bonferroni correction), identifying significant characters in the patient’s IDPN. Finally, using the aforementioned method, we constructed IDPNs for each patient (participant with NSSI). Each IDPN consists of 136 characters between 17 features.

### Subtyping NSSI individuals using IDPN characters

After establishing the IDPN, we first calculated the number of characters showing significant changes in each character among all patients (*p* < 0.05, Bonferroni corrected). Subsequently, we selected 8 characters as clustering variables for K-means clustering analysis. The rationale for choosing these 8 characters is that they exhibited significant changes in at least 2% (*n* = 14) of the samples. Before clustering, the variables required for clustering were transformed into Z-standardized scores [43], and the clustering analysis by SPSS was performed using these standardized values.

## Results

### Demographic information

In this study, a total of 2,376 participants were included, with a mean age (*SD*) of 13.66 (0.98). Females comprised 52.2% of the total sample (*n* = 1,240), with a mean age (*SD*) of 13.62 (0.99). Males comprised 47.8% of the total sample (*n* = 1,136), with a mean age (*SD*) of 13.70 (0.98).

Among the overall sample, 881 participants experienced NSSI, resulting in a prevalence of 37.1%. Among those who reported NSSI, the mean age (*SD*) was 13.61 (1.02). Females accounted for 56.4% (*n* = 497) of the NSSI sample, with a mean age (*SD*) of 13.58 (1.04). Males accounted for 43.6% (*n* = 384) of the NSSI sample, with a mean age (*SD*) of 13.66 (0.99).

In addition, we also calculated the mean and *SD* of each scale in the sample (Table S1).

### Clustering results by K-means

We used the Elbow method to determine that the optimal number of clusters for K-means analysis is 2 (Figure S1). After 40 iterations, we obtained the current clustering results: Group 1 contained 560 patients, and Group 2 contained 321 patients. Collinearity checks showed that the Variance Inflation Factor (VIF) for each variable was less than 3, indicating no collinearity among variables. Subsequently, we validated the clustering effect using Fisher’s discriminant analysis [44], which showed a classification accuracy of 95.8%, indicating good clustering accuracy.

### Difference of individual network characters in two groups

**Fig. 2:**
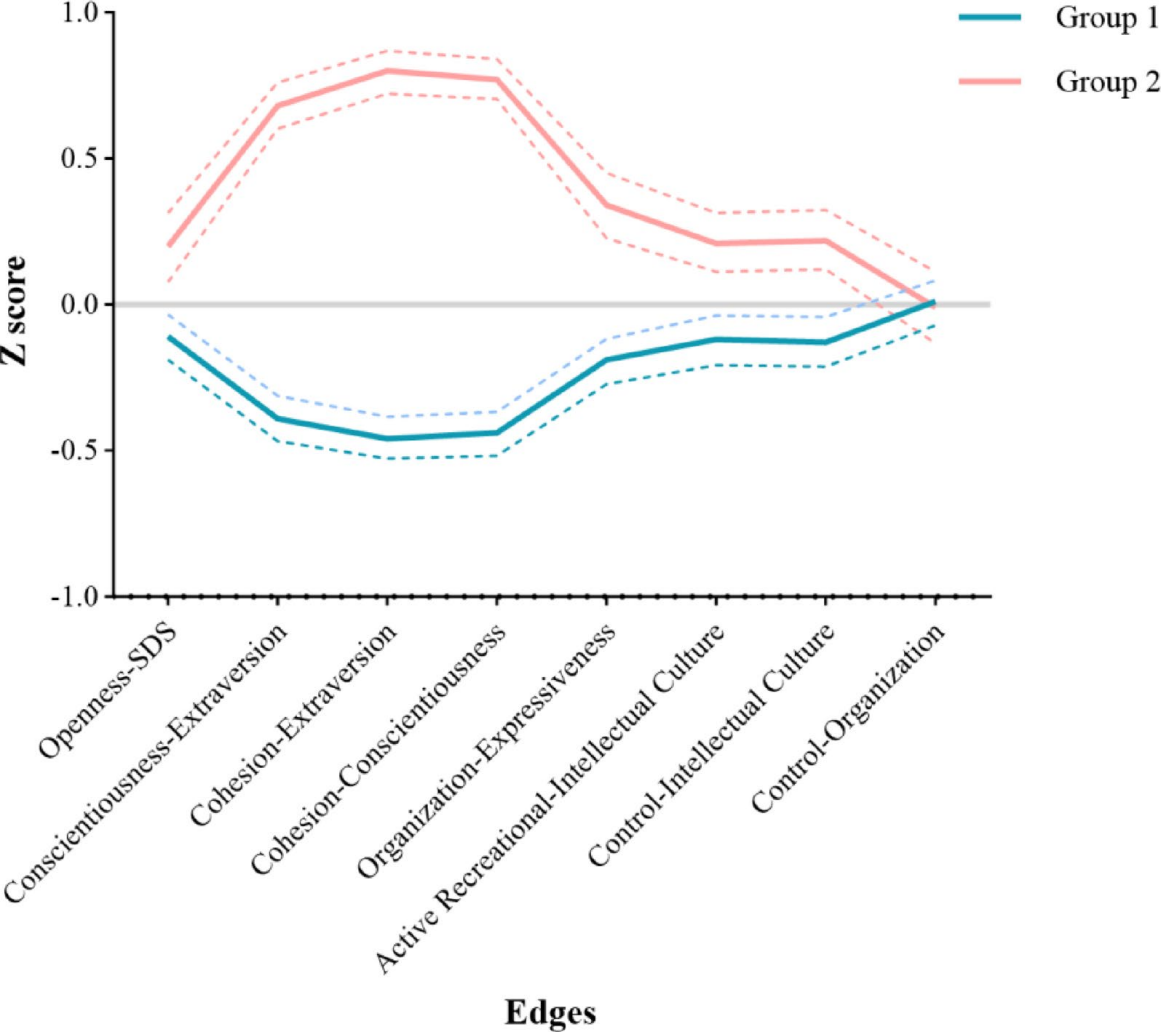
The mean Z-score of the characters in both groups. Note: Dashed lines represent the 95% CI of the Z scores for each character

**Fig. 3:**
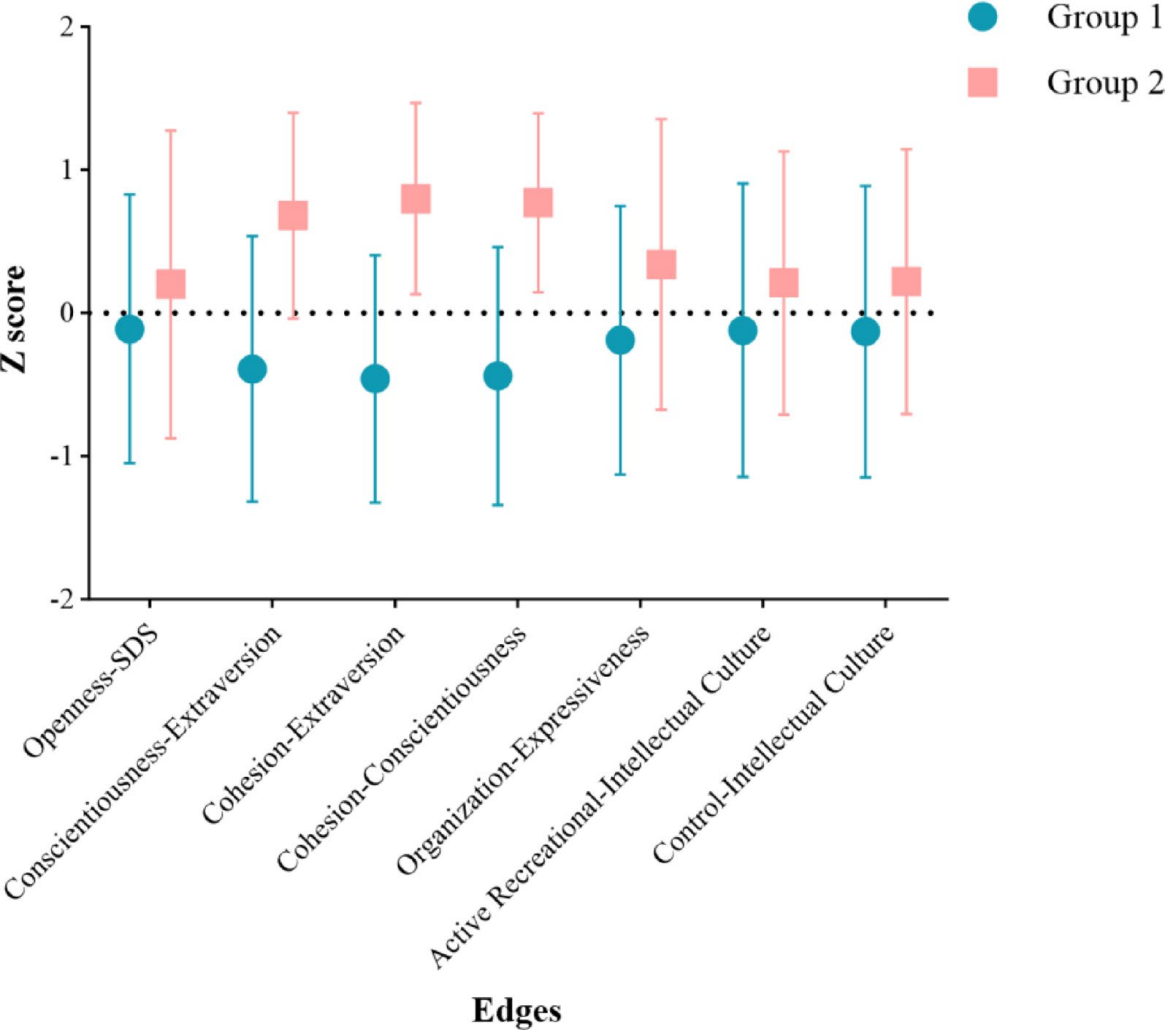
The mean and standard deviation (***SD***) of significantly different characters between the two groups. Note: Error bars represent the standard deviation of the Z scores for each character

We plotted the mean Z scores and their 95% confidence intervals (*CI*) for the selected 8 characters, and observed that except for “Control-Organization,” the values of the other 7 characters (“Openness-SDS”, “Conscientiousness-Extraversion”, “Cohesion-Extraversion”, “Cohesion-Conscientiousness”, “Organization-Expressiveness”, “Active Recreational-Intellectual Culture”, “Control-Intellectual Culture”) in Group 1 were lower than those in Group 2 (Fig. 2). Upon conducting *t*-tests of the 8 characters between the two groups, we found no significant difference in “Control-Organization” (*p* > 0.05), while significant differences were observed of the other 7 characters (*p* < 0.05, Table 1). Specifically, the *Z*-scores in Group 1 were consistently lower than those in Group 2 (Fig. 3).

**Table 1:** Comparison of differences in characters between the two groups

	Group 1 mean Z-score (*SD*)	Group 2 mean Z-score (*SD*)	*t*	*P*	Cohen’s d
Openness-*SD*S	−0.11 (0.94)	0.20 (1.07)	−4.28	< 0.01	−0.31
Conscientiousness-Extraversion	−0.39 (0.93)	0.68 (0.72)	−19.09	< 0.01	−1.25
Cohesion-Extraversion	−0.46 (0.86)	0.80 (0.67)	−24.00	< 0.01	−1.57
Cohesion-Conscientiousness	−0.44 (0.90)	0.77 (0.62)	−23.56	< 0.01	−1.50
Organization-Expressiveness	−0.19 (0.94)	0.34 (1.01)	−7.72	< 0.01	−0.55
Active Recreational-Intellectual Culture	−0.12 (1.02)	0.21 (0.92)	−5.00	< 0.01	−0.34
Control-Intellectual Culture	−0.13 (1.02)	0.22 (0.93)	−5.21	< 0.01	−0.36
Control-Organization	0.01 (0.93)	−0.01 (1.11)	0.22	0.83	0.02

Note: The mean *Z*-scores of the 8 characteristics between the two groups, and the results of the *t*-test comparing the intergroup differences

### Difference of NSSI severity in two groups

**Fig. 4:**
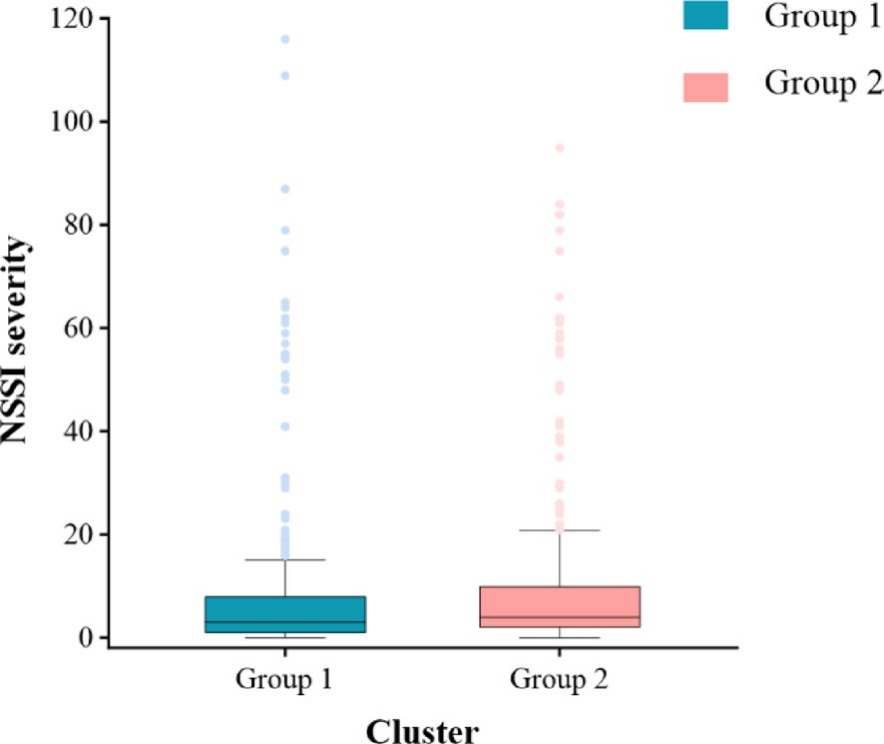
Difference of NSSI severity between the two groups. Note: Error bars represent the 10th to 90th percentile score distribution range of NSSI severity

We compared the NSSI severity between the two groups and found that the severity in Group 1 was lower than in Group 2 (*p* < 0.05). Further observation revealed that although the maximum NSSI severity in Group 1 (116) exceeded that in Group 2 (95), the first quartile, median, third quartile, and the 10th to 90th percentile score distribution range in Group 1 were all lower than those in Group 2. This validates our t-test results, indicating that the overall NSSI severity was lower in Group 1 (Fig. 4 and Table S2).

## Discussion

This study pioneers the application of individual network construction methods in the field of psychological measurement, using individual network approaches to explore clinical outcomes from the perspective of correlations among psychological features. Our study results primarily highlight three points: (1) Among individuals with NSSI, there is low similarity in IDPNs across individuals; (2) The 8 selected characters primarily involve depression, personality traits, and family environment, with anxiety not included; (3) Using IDPN characters, NSSI cases were classified into 2 groups, with Group 1 showing lower NSSI severity and psychological feature correlations compared to Group 2. This method can help us to further explore the correlation between individual features and help to identify different patient subgroups.

In our study, we found that the 8 selected characters were unrelated to symptoms of anxiety. While numerous previous studies have demonstrated the relationship between anxiety and NSSI [45, 46], some have presented contrasting results, suggesting that anxiety may not consistently serve as a stable risk factor for NSSI [47]. We hypothesize two potential reasons for this discrepancy: Firstly, the impact of anxiety on NSSI is often overshadowed by depression [17]. Our previous mediation analysis on anxiety, depression, and NSSI indicated that individual anxiety may lead to depression, which in turn contributes to NSSI [17]. Although some studies have reported that 30.9% of individuals with NSSI exhibit symptoms of anxiety and 36.9% of those with anxiety engage in NSSI [47], these figures do not significantly differ from those observed in the general population [48–50]. Therefore, the exact influence of anxiety alone on NSSI warrants further investigation.

Secondly, we constructed IDPN based on correlations among psychological features rather than the features themselves. The relationships between anxiety, depression, personality traits, and family environment with NSSI have been extensively documented [51, 52]. However, low correlations among psychological features may indicate that each feature plays a relatively isolated role in the development of NSSI, without triggering a cascade of reactions and thus potentially being more amenable to intervention. Conversely, high correlations among psychological features suggest close interconnections, where targeting a single feature in isolation may not yield optimal results. Prior research has also supported this notion: intervening in closely linked networks of disease symptoms may easily lead to disease relapse [53].

In this study, children and adolescents with NSSI were divided into two groups. Group 1 exhibited significantly lower NSSI severity compared to Group 2, and the scores of characters in Group 1 were lower than those in Group 2 (except for Control-Organization). Our findings indicate that the severity of NSSI is closely associated with family environment, personality traits, and depression, and the closer the relationships between these features, the higher the NSSI severity.

Individuals engaging in NSSI often share similar personality traits. Some studies based on Cloninger’s personality theory [54, 55] have found that individuals with NSSI tend to score higher on Novelty Seeking (NS) [56] and Harm Avoidance (HA) [57], while scoring lower on Self-Directedness (SD), Cooperativeness (C), and Persistence (P) [58]. Other explorations into NSSI and personality traits have shown higher scores in neuroticism [51] and impulsivity [59] among individuals with NSSI, while scores in self-control are lower [59]. Individuals with borderline personality disorder (BPD) exhibit a higher intensity of NSSI [60], and a higher proportion of individuals engaging in NSSI are diagnosed with BPD [61]. The influence of personality traits on NSSI may operate through their impact on emotion regulation strategies [62], as current research suggests that NSSI arises from emotional dysregulation [63] and impaired impulse control [64].

In terms of family environment, individuals engaging in NSSI tend to score lower in family cohesion and family adaptability [65]. Many other studies have also identified various aspects of family environment that influence NSSI occurrence, such as high family stress, poor parent-child relationships [66], low levels of family psychological support [67], and parental separation [68]. The NSSI Family Distress Cascade Theory suggests that adolescence is a high-risk period for family issues, possibly due to the dynamic tension between individual autonomy and family support during this developmental stage, influenced by factors such as hormone levels and emotional behavior [69]. NSSI serves as a coping mechanism, and when faced with unresolved family problems, children and adolescents may resort to NSSI. As mentioned in the NSSI Family Distress Cascade Theory, many adolescents view NSSI as a symbol of autonomy [19]; they often conceal their NSSI behaviors from parents, who may suspect something is wrong based on their emotional changes but are usually unaware of the specific actions taken by their kids. Caregiver suspicions further intensify the secrecy surrounding NSSI behaviors among adolescents, creating a cycle where caregivers seek to uncover the truth while adolescents strive to conceal their actions, worsening family relationships and exacerbating the frequency and severity of NSSI among adolescents [69].

NSSI is significantly associated with depression [31] and can predict the occurrence of NSSI [70]. Additionally, individuals engaging in NSSI are prone to various emotional disorders such as depression [71]. There are several reasons for the close relationship between NSSI and depression. Firstly, NSSI serves as a means or strategy to alleviate negative emotions [72], implying that individuals engaging in NSSI may already have a series of underlying emotional issues before the onset of NSSI. The exacerbation of these emotional issues increases the risk of NSSI, which subsequently occurs. Secondly, NSSI often recurs [73], causing dual physical and psychological harm to patients. Scars left on the body after self-injury may also attract unwanted attention from others, making individuals engaging in NSSI more prone to emotional issues [17]. Thirdly, depressive symptoms are closely linked to aggressive behavior [74], and NSSI is one manifestation of such behavior. Finally, existing research has found associations between NSSI and dysregulation of the hypothalamic-pituitary-adrenal axis [75, 76], a pathway implicated in theories of depressive disorders. Thus, abnormalities in hormone levels within shared pathways may play a role in the occurrence of NSSI and depression.

There is also a close connection between personality traits, family environment, and depression. Patients with depression often exhibit high neuroticism, low extraversion, and low conscientiousness [77], and they tend to experience more family dysfunctions, such as increased parent-child conflicts and poorer family communication [78]. Regarding the relationship between family environment and personality traits, the family environment is the primary external environment during the growth of children and adolescents, closely related to their psychological development and the formation of their personality traits [79, 80]. The close relationship among these three features forms an influence network that further impacts NSSI. Moreover, the close connections among these features also partially validate the correctness and effectiveness of the characters identified in our IDPN.

Constructing the IDPN based on individual differences offers clear advantages for exploring features at the individual level. Moreover, clustering based on IDPN features facilitates the investigation of the heterogeneity of psychological characteristics in NSSI patients, providing novel feature indicators for future studies. Nevertheless, several inherent limitations of IDPN should be acknowledged. First, when selecting the most influential characteristics, there is currently no standardized criterion; choices are largely empirical and depend on the properties of the data [28, 40]. Second, the construction of reference and perturbed networks, along with the subsequent series of computations, involves substantial computational demands, placing high requirements on computational resources. Third, while IDPN offers new perspectives and feature indicators whose scientific validity has been demonstrated, directly linking these computed metrics to clinical interpretation remains challenging. For instance, in the present study, we observed that the correlation between two specific features had a significant impact on NSSI, suggesting a potential relationship between these features and NSSI. However, how such relationships can be translated into concrete guidance for clinical practice requires further investigation. Even so, IDPN, as an individual-level heterogeneity analysis method, provides a valuable new perspective that can be further explored in future research.

Moreover, we observed an interesting phenomenon: anxiety did not appear among the most influential characters. There are two possible explanations for this. First, the IDPN constructs a differential network, with differences calculated between NSSI individuals and non-NSSI controls. If anxiety does not differ substantially between these two groups, it may not emerge as an influential feature. Considering that all participants in our sample were students and that anxiety is common during adolescence [81], this observation is plausible. Second, in selecting the most influential characters, we applied a 2% threshold. Compared with thresholds used in previous studies [28], our criterion was slightly more stringent, which may have overlooked features with smaller, yet still meaningful, effects.

This study has certain limitations. First, the data used in this study were all from self-report questionnaires, which may be subject to participants’ concealment. Second, the data used in this study were cross-sectional, which prevents any inference about the time course or directionality of the observed relationships. Third, in this study, the reliability of the FES-CV reached an acceptable level (Cronbach’s alpha > 0.6) [82, 83]; however, the overall reliability was still relatively low, which may be attributed to the large number of items and the use of a secondary rating scale. Fourth, including more influencing factors of NSSI in future studies is necessary, as this would not only allow for validation of the stability of the IDPN approach but also account for potential interactions among these factors. Fifth, the discriminant function for validating the K-means clustering was applied to the same set of samples. This approach may slightly overstate the classification accuracy. Future studies could use cross-validation to provide a more reliable assessment of the discriminant performance. Finally, to our knowledge, this is the first study to apply the statistical methods of IDSCN to psychometric data, so further research is needed to verify the extensibility of our findings. In the future, IDPN should be further validated in different samples or across different disorders. Such validation could incorporate longitudinal samples, more reliable and objective assessment tools and metrics, as well as additional influencing factors to improve and refine the validation of IDPN. However, our introduction of the concept of IDPN is a strength of our study, as it may provide new research features for the field of psychometric research.

## Conclusion

We have extended the theory of individual differential networks to the field of psychometrics for the first time and proposed the concept of IDPN. Based on our findings, we found that IDPN can help further explore the relationships among individual characteristics and assist in identifying distinct patient subgroups. However, it should be noted that IDPN is still in its early stages, and further research is needed to validate its reproducibility and generalizability.

## Supplementary Information

Supplementary Material 1.

Supplementary Material 2.

## Data Availability

The datasets generated and analyzed during the current study are not publicly available due to privacy or ethical restrictions but are available from the corresponding author on reasonable request.
