# Supplementary material for: A novel approach to exploring youth non-suicidal self-injury heterogeneity: individual differential psychopathology network analysis
Source: Ann Gen Psychiatry. 2025 Oct 17;24:63. doi: 10.1186/s12991-025-00606-5 (PMC12535082; doi:10.1186/s12991-025-00606-5)
Supplement: Supplementary file 1 — Supplementary Material 1. [file 12991_2025_606_MOESM1_ESM.docx]

Table S1. Score statistics of each scale

|  | Total sample | Boys | Girls |
| --- | --- | --- | --- |
| Number (%) | 2376 (100%) | 1136 (47.8%) | 1240 (52.2%) |
| Number of NSSI（%） | 881 (100%) | 384 (43.6%) | 497 (56.4%) |
| SAS score (*SD*) | 41.92 (8.978) | 40.42 (8.116) | 43.30 (9.496) |
| SDS score (*SD*) | 46.85 (11.182) | 45.25 (10.273) | 48.31 (11.770) |
| Neuroticism (*SD*) | 34.66 (4.270) | 34.11 (4.407) | 35.17 (4.076) |
| Extraversion (*SD*) | 37.64 (4.177) | 37.54 (4.286) | 37.73 (4.075) |
| Openness (*SD*) | 33.32 (4.898) | 33.51 (4.869) | 33.15 (4.919) |
| Agreeableness (*SD*) | 34.46 (4.004) | 35.04 (4.100) | 33.93 (3.841) |
| Conscientiousness (*SD*) | 37.46 (4.268) | 37.54 (4.567) | 37.38 (3.976) |
| Cohesion (*SD*) | 7.37 (2.065) | 7.54 (1.813) | 7.22 (2.261) |
| Expressiveness (*SD*) | 4.94 (1.656) | 4.96 (1.515) | 4.92 (1.776) |
| Conflict (*SD*) | 2.74 (2.140) | 2.42 (1.959) | 3.03 (2.254) |
| Independence (*SD*) | 4.96 (1.482) | 5.02 (1.491) | 4.92 (1.472) |
| Achievement (*SD*) | 4.54 (1.419) | 4.54 (1.400) | 4.53 (1.436) |
| Intellectual-Cultural (*SD*) | 4.06 (2.117) | 4.07 (2.137) | 4.05 (2.100) |
| Active-Recreational (*SD*) | 4.72 (2.510) | 4.82 (2.378) | 4.64 (2.634) |
| Moral-Religious (*SD*) | 5.66 (1.431) | 5.76 (1.424) | 5.57 (1.432) |
| Organization (*SD*) | 6.16 (1.971) | 6.24 (1.819) | 6.08 (2.099) |
| Control (*SD*) | 3.57 (1.977) | 3.54 (1.969) | 3.61 (1.985) |

Table S2. The scores of NSSI severity in both groups and the overall sample.

| NSSI severity | Mean | *SD* | *p* (*t*-test) | 25% | 50% | 75% | Min | Max |
| --- | --- | --- | --- | --- | --- | --- | --- | --- |
| Group 1 | 7.18 | 12.924 | 0.024 | 1 | 3 | 8 | 0 | 116 |
| Group 2 | 9.47 | 15.283 |  | 2 | 4 | 10 | 0 | 95 |
| Total | 8.01 | 13.866 | / | 1 | 4 | 9 | 0 | 116 |

Note: The mean, *SD* (standard deviation), interquartile range, maximum, and minimum of NSSI severity for the two groups. Used t-test to compare the intergroup differences in NSSI severity.
