# Supplementary figures and images for: A novel approach to exploring youth non-suicidal self-injury heterogeneity: individual differential psychopathology network analysis
Source: Ann Gen Psychiatry. 2025 Oct 17;24:63. doi: 10.1186/s12991-025-00606-5 (PMC12535082; doi:10.1186/s12991-025-00606-5)

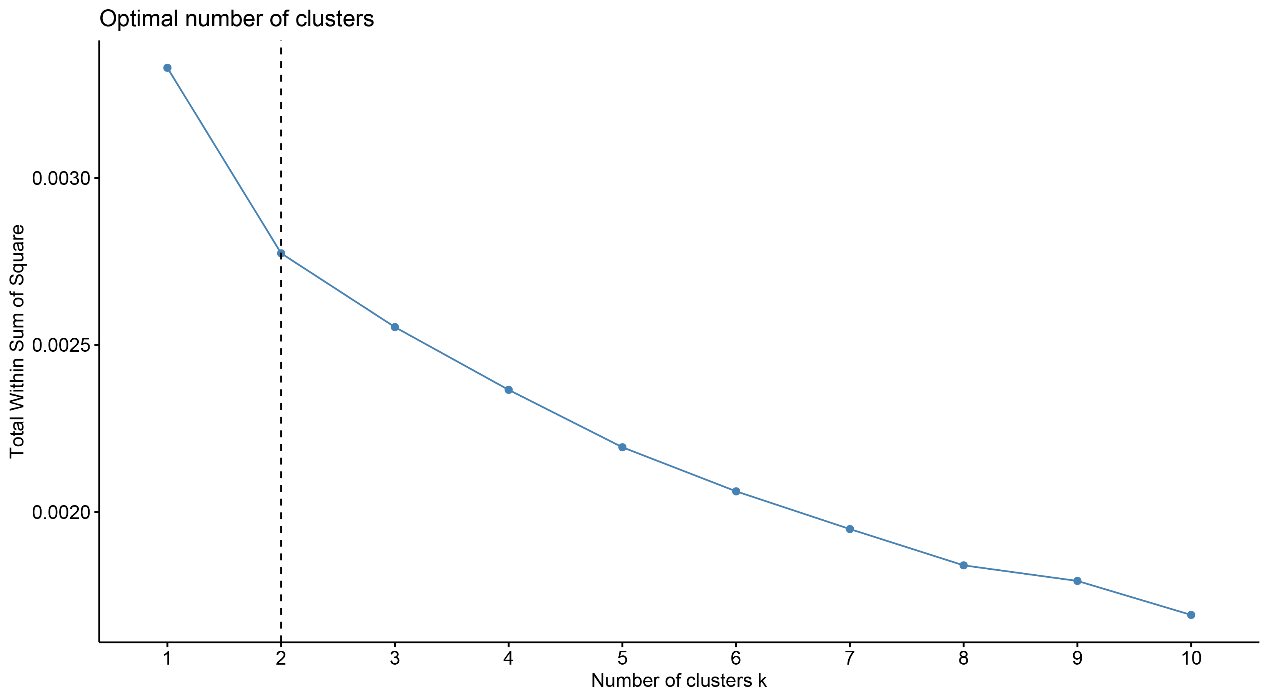


Figure S1. Result of the elbow method for determining the number of clusters

Supplement: Supplementary file 2 — Supplementary Material 2. [file 12991_2025_606_MOESM2_ESM.docx]
